# Monthly Summary

**Published:** 1868-08

**Authors:** 


### MONTHLY SUMMARY.

---

*Case of Foreign Body in the Œsophagus.*—By Lewis A. Sayre, M. D.

Charles M'C—, aged 19, living at the St. Nicholas Hotel, between midnight and one a. m. of the morning of 29th April last, waked and found himself in the act of swallowing the plate to which was attached an artificial right central incisor tooth which he was in the habit of wearing. He endeavored to seize it, but the power of the muscles of deglutition was so great that he lost his hold, and the plate passed into the œsophagus. Without waiting to dress he ran to the house of a physician in Broome Street, who attempted to dislodge the obstruction by passing a sponge probang, but without effect beyond causing slight hæmorrhage. He then took an emetic, which also failed to remove the plate. About 3 a. m. he went to the New York Hospital. Dr. Wainwright, one of the house surgeons, with an assistant, examined his throat with care, both with a probang and œsophageal forceps, and not feeling any foreign body, naturally inferred that the pain in his throat was due to the repeated explorations to which he had been subjected. Dr. Wainwright informs me that he passed the forceps a number of times, but was never fully satisfied that he felt any foreign body, although the patient insisted that the tooth was in his throat, and begged that it might be cut out. On the 30th, forty-two hours after the accident, he presented himself at my office. His countenance was anxious and pale; the paleness and debility being explained by the fact that he had been unable to swallow anything since the accident. The head was thrust forward, and fixed in a stiff and awkward position, the respiration had a peculiar wheezy character, the voice was husky and rasping. On either side of the neck, just behind the sterno-cleido-mastoid muscles, and about an inch below the thyroid cartilage, a slight fullness could be detected; pressure upon these points produced a sense of suffocation, and placing his finger just above the sternum, he said the tooth was cutting him at that point.

I passed a whalebone probang down the œsophagus without obstruction, and also a pair of curved forceps, and could feel no foreign body. I again pressed his neck, and he stated that the tooth cut him "just there," putting his finger at the point just above the sternum before mentioned, and that the "hook" on the plate was caught "just here," putting his finger on the right side of his neck. I then asked him if he could describe the plate or make a drawing of it, when he made a rough diagram, which gave me a very good idea of the size and shape of the body in the œsophagus. It also explained the position of the plate in the throat, and I at once perceived that while the œsophagus was distended by the plate extending from side to side, the probang and forceps had readily passed through the concavity of the plate, and had been withdrawn without touching the latter.

I therefore determined to use the *bristle probang.* This instrument was invented I think by a surgeon in the East India service, but whose name I never heard. For an emergency occurring some weeks previously, I had made one of these instruments in the following manner. I took an ordinary No. 10 elastic catheter and cut off about one inch from its lower end, I then ran through it a flexible whalebone about three inches longer than the catheter, and tied on its end a small piece of sponge. I then took to pieces an ordinary paint brush, and tied one end of the bristles around the sponge, completely surrounding the whalebone rod with them. The other end of the bristles I tied around the cut extremity of the catheter.

By a slight twisting, the bundle of bristles can be reduced in diameter to about the size of the catheter. By holding the catheter firmly with one hand, and with the other drawing upon the rod so as to bring the sponge toward the catheter, each of the bristles is bent into a loop, and the whole bundle is converted into a disc about five inches in circumference, large enough to completely sweep the œsophagus and to remove any foreigh body lodged therein.

The sponge having been dipped in water, the instrument was extended, and was readily passed the entire length of the œsophagus without obstruction; it was then distended in the manner described, and slowly withdrawn with a slightly twirling movement, so as to sweep all parts of the tube, and fortunately brought out the plate and tooth riding on its meshes without difficulty, and with scarcely any pain.

The plate had at either extremity a hook fitting to the teeth for its retention in the mouth, besides a number of smaller points and ridges for the same purpose. Its dimensions were about two and a quarter inches from hook to hook, by one half inch wide. The tootn and gum which projected above and below the plate at right angles to it was about five eights of an inch in length by one quarter in width

The lad had some tenderness in swallowing for a few days, which soon passed away.—*New York Medical Journal.*

*Divisibility of Matter.*—Gold can be beaten into leaves ·00004 of a millimeter thick.

Silver wire, gilded with ·00277 of its diameter of gold, can be drawn so fine that one meter weighs only eight milligrammes. The gold film of this wire is now only ·00000125 of a millimeter thick. By placing a short piece of this wire in nitric acid, the silver core is dissolved out, leaving a tube of gold, having a wall only the ·00000125 millimeter thick. Now under the best microscopes, we can discern a surface of ·00025 millimeter in diameter, and ·0000012 millimeter thick; yet each of these parts has all the physical and chemical properties of a large mass, as can be determined by testing it under a microscope.

Dr. Wollaston drew platinum wire so fine that its diameter was only ·000833 millimeter (000033 of an inch:) and although platinum is the heaviest of the metals, yet it took 200 meters of this wire to equal one centigramme in weight; or, in other words, one mile of this wire weighed about one grain. Dr. Wollaston accomplished this by wire drawing a cylinder of silver ·2 of an inch in diameter, having in its axis a platinum wire ·01 of an inch in diameter, and after having obtained a very fine wire—having in its interior a platinum wire of still greater tenuity—he dissolved with nitric acid the silver coating, and thus obtained an almost invisible platinum wire.

The thickness of a soap bubble, in the dark spot which is formed on it just before it bursts, is ·00001 millimeter.

One grain of carmine tinges ten pounds of water, which we can divide into about 617,000 drops. If we suppose that 100 particles of carmine are requisite to produee a uniform tint in each drop, it follows that one grain of carmine has been divided into 62,000-000 parts.

The thread of a spider is composed of more than 1,000 separate threads.

The diameter of the red disks contained in human blood is ·00025 of an inch; while the diameter of the blood disks of the Java musk-deer is only the ·000081 of an inch, so that a drop of this deer's blood, such as would adhere to the point of a fine needle would contain 150,000,000 disks.

It has been calculated that some of the siliceous plates which cover and give rigidity to the minute vegetable cell-plants called diatomaceæ, weigh only the ·0000000005 of a grain yet the surfaces of these plates are covered with the most exquisite tracery of siliceous stria or bars, often not more than ·0000117 of an inch in width and thickness. Now we can discern a surface of ·0000117 of an inch in the best microscopes, and a portion of one of these siliceous disks of that area would weigh only about ·00000000002222 of a grain.

A portion of musk will give off a powerful odor during a year, and yet its diminution in weight has not been sufficient to be detected by the most delicate balance.

"In order to offer an inexact idea of the minuteness of the particles of musk which are still capable of imparting some odor, we state, after a well known experimenting physiologist, that a certain liquid, containing as much of an extract of spirit of musk as ·000000005 part of its whole weight, was at this time still distinctly odorous. A grain's weight of a liquid of which ·0000005 part was of that extract, spread an intensely penetrating odor. Next after musk are to be mentioned certain flower ethers, especially the oil of roses, a little drop of which is sufficient to fill with odor an immense atmosphere. The same physiologist states that a certain space filled with air, of which, at the highest, only ·000001 part was vapor of oil of roses, still diffused a distinct odor of roses."—*Prof. Mayer.—Scientific American.*

---

*Sponge Tents.—Carbolized.*—Sponge tents may be rendered incapable of putrefaction by introducing into the core of the tent several threads of cotton wick steeped in carbolic acid and after the sponge is rolled into its proper shape, by immersing it into cocoa butter to which a certain quantity of glacial carbolic acid is added. The disinfectant properties of this agent completely protect the tents, and they are withdrawn in an inodorous state even after a stay of eighteen or twenty hours in the cervical canal.—*Braithwaites Retrospect.*

---

*Spray of Æther for Relieving Pain.*—Dr. Horaud, of Lyons, France, reports very favorably on this remedy. Although the influence of nebuilzed æther may not be permanently curative, it certainly is very valuable as a means of affording at least temporary relief in severe local pains, and especially in cases of neuralgia, the pain of which is at times so fearfully distressing.—*Jour.de Med. de Lyon.*

*Staphyloraphy.*—Robt. S., æt. 18, from Ohio. This patient came to Prof. Gross on account of cleft in the palate, congenital in character, as it generally is, involving simply the soft parts. He was operated on last Friday a week ago. The edges of the fissure were pared, making raw surfaces of them, precisely as in the operation for hare lip, and then the parts were approximated with five twisted sutures, retained by means of shot. There was then introduced between two of these sutures an ordinary interrupted suture. Perfect union has been obtained with the exception of a little point toward the apex of the cleft, so small as hardly to be visible. The resulting inflammation was not more than was expected and desired. The interrupted suture was removed at the end of the fifth day, the others at the end of the eighth. The edges of the surface were touched with a weak solution of nitrate of silver, ten grains to the ounce, and yesterday and to-day with the solid stick of nitrate of silver.

The little opening left seems to be perfectly round, and if still remaining can be readily closed by the introduction of another suture next October, as he is anxious now to go home.

The patient was fed abundantly after the operation which is a matter of great importance. He is now taking exercise in the open air, and will probably go home next Friday, He has been able to swallow well ever since the operation. It will be a good while before there is much improvement in speech, which can only be effected by daily, constant instruction and practice in the pronunciation of the alphabet.—*Clinic of Prof. Gross Med. & Surg. Reporter.*

---

*Poisons Found in Snuff*—Prof. Vander Weyde says that the most common adulteration in snuff, made from inferior insipid kinds of tobacco, are quick lime or some caustic alkali; the latter substances serve a quadruple purpose; 1st, of developing an acrid and pungent effect on the olfactory organs; 2d of keeping up a desirable degree of moisture; 3d, increasing its weight, it being always sold by weight; 4th, neutralizing the acid developed by the fermentation of the molasses and water which enters in the regular sauces, added at an early period of its preparations.

For the purely pungent effect, sometimes very finely powdered glass is added, for giving it a peculiar flavor, urine, chloride of ammonium, and sometimes rum are used, for keeping it moist, common salt with chloride of calcium or magnesium, and more recently glycerine has been introduced.

The above substances are easily detected in common kinds of cheap snuff, by any chemist who will take the pains to search for them, but it must not be supposed the more expensive kinds are better in this respect, for instance th Scotch, Spanish, Russian, Maccoby, Welsh, Lundy foot, French, Strasburg snuffs, are mostly medicated, either to give a peculiar flavor or color, or some remedial virtue or vice. These substances are nitrate of silver, the turpeth mineral or basic sulphate of mercury, or red sulphide of antimony, tartar, emetic, etc. In this way, they obtain undoubtedly some remedial effect in cases of neuralgia, opthalmia, migraine, tic douloureux, etc.

However, it is almost unnecessary to remark, how the medical practitioners may be annoyed by symptoms produced by overdoses of such drugs, introduced in the system by an unsuspected channel.—*Med. & Surg. Reporter.*

---

*Effects of Darkness and Silence.*—Dr. Kane and other Arctic voyagers have all testified that, in those regions "where eternal silence reigns supreme," the effect upon the brain and ear from the absence of sonorous impulses in the atmosphere is exceedingly annoying and absolutely injurious to the auditory nerves. As the organs of hearing are destroyed by loud and continued noise, and an intense light will weaken and ultimately destroy the power of sight, so it would appear that the auditory or optic nerves become impaired by the partial or total deprivation of their natural stimulus, sound or light. Dr. H. Ralls Smith, of Chicago, wishing experimentally to investigate this subject, recently spent a considerable length of time in the Kentucky Mammoth Cave, where silence and impenetrable darkness reigned supreme. The effect was very distressing, and almost insurmountable, resulting in temporary defection of hearing and aberration of mind. From his own experience, this gentleman is firmly convinced that the blindness of the finny denizens of this cave has been brought about gradually through successive generations, and from his observations he is confident that the sense of hearing is also wanting in these beings, although originally existing in the species when first immersed in their living tomb.—*Scientific American.*

*Early Trials of Experimental Physiologists*—M. Claude Bernard in his recent "Report on the Progress of General Physiology in France," gives the following as his experience when he began to teach physiology experimentally, an experience which we doubt not is similar to that which physiologists have gone through in this country.

"When, some twenty-five years since, I entered upon the career of experimental physiology, I found myself subjected to all the annoyances which were the fate of experimenters. At that time it required to be sustained by a true love for physiology, and to possess patience and courage, often very considerable, in order to keep one's position. As soon as an experimenting physiologist was discovered he was denounced, voted as abominable by his neighbors, and subjected to the pursuits of the police."

Analyzing the report, the *Lancet* says: "Strangely enough, on one occasion M. Bernard found his best protector was a police commissaire. Having introduced a silver tube into the stomach of a dog, in order to demonstrate to Dieffenbach, who then happened to be in Paris, Blondot's experiments on the gastric juice, the brute escaped from the room in which he was confined, carrying the tube still projecting from the stomach. A few days afterward the experimenter was sent for by a police commissaire, who, showing him a dog, demanded whether he was the person who had introduced the instrument into his abdomen. He replied in the affirmative, adding that he was delighted to recover the silver canula, wh ch he had given up as lost. This threw the commissaire into a passion, who demanded how he had dared to take his dog as the object of his experiments. He explained to him that the dog had been bought from persons who were supposed to be authorized by the police to sell unclaimed dogs, and that the animal would do very well. In fact, M. Bernard went daily to see the dog until the wound was healed, and so ingratiated himself in the favor of the commissaire and his family that he found him, for several years after, a most efficient protector against all denunciations and interruptions of his investigations."—*Med. Gazette.*

---

*Preservation of Vaccine Virus by Glycerine*—Geheimer Rath Muller, Director of Vaccination at Berlin, observes in a recent letter, that he had hoped it would not be necessary for him to write any more concerning the advantages of vaccine virus diluted in glycerine. Finding, however, that some prejudices and ignorance still obtain on the subject, and in presence of an epidemic of variola now prevailing in Prussia, where, in many places, practitioners find it impossible to obtain virus in sufficient quantities for revaccination, he feels it incumbent on him to state that a two years experience in the use of his glycerined virus justifies his warmest recommendation of its employment, and that some lymph thus preserved for nearly two years, still retains its efficacy. Of course, those who by carelessness have mixed lymph unfit for use with glycerine, or have mixed good lymph with impure glycerine, may well have been disappointed in the results obtained. He adds, that, on the breaking out of variola in the prisons, two thousand persons were immediately revaccinated, which could never have been done in the month of February without the stores of glycerined lymph supplied by the establishment. Moreover this rapid revaccination arrested at once the progress of the epidemic.—*Medical Times and Gazette.*

---

*Odontomes.*—M. Broca, the distinguished surgeon and physiologist, has just elucidated the pathology of the follicles of the teeth, the normal evolution of which had already been described in works on histology. M. Broca does not think that the deviations from this normal evolution give rise to peculiar products, but only to tumors made up of the general hypertrophy of the dental substance. These tumors, to which the author gives the name of "odontomes," present two forms: some always remain in the state of more or less soft tumors; whilst others, either wholly or in part, assume the hardness of teeth, producing shapeless, irregular dental masses, sometimes growing to a very large size. In fact, any tumor formed from one or more of the substances entering into the formation of a tooth, is due to the dentification of a soft tumour of the same form and volume which originally contained only hypertrophied odontogenic tissues. This hypertrophied tumor stands in the same connection with regard to the dentified tumor, as the normal dental bulb does to the healthy tooth.—*Lancet.*

*Chloroform to Cure Sneezing*—Some persons are subject to paroxysms of sneezing, which come off without manifest cause, and occasion much annoyance and sometimes no small degree of exhaustion. The best remedy, and in fact the only one we know of, is chloroform,the vapor of which must be snuffed up the nostrils. A few snuffs from the mouth of the vial will often suffice. Whether the same remedy would prove effectual in the very often distressing and exhausting sternutation of "Hay Asthma" we have no opportunity to determine. It is worthy a trial, however.—*Pacific Medical & Surgical Journal.*

---

*Singular Cause of Death.*—A London Coroner recently confirmed a very singular cause of death. The subject was a widow, who died after long suffering. The report of the physician charged with the inquest, establishes the fact that the death was caused by poisoned goose-grease. It would seem that cases of such empoisonment are not unfrequent.. The poison is produced in the fat of this bird by different causes; first, by decomposition of the flesh, when life has been long extinct, and next by the food absorbed by it, particularly by poisonous plants. This nourishment engenders and concentrates in their grease a very dangerous poison.—*L' Evenement Medicale.*

---

*The Use of Veratrine in Neuralgia.*—Hector Bertrand testifies to the efficacy of veratrine in neuralgia, particularly of the trigeminu nerve. He employs an ointment composed of from 30 to 40, or even 50 centigr. of veratine, 25 centigr. of morphia, and 30 grammes of lard. This is rubbed along the course of the affected nerve. In 5 to 10 minutes a gradually increasing feeling of warmth, accompanied by a crawling sensation, (tingling and numbness) is felt, without apparent alteration of the cutis. As this feeling increases, in about 15 or 20 minutes, the pain loses its activity. A cure is commonly effected in three or four repetitions of the frictions.—*Recueil de Mem. de Med., etc.*

---

*Lancing the Gums in Children.*—Dr. F. H. Thomson, believing that the irritation of teething is caused by the engorgement of vessels supplying the circulation, advises the practitioner to cut low down at the reflected junction between the lip and the gum, instead of upon the summit of the gum itself.—*Med. Record.*

*A New Styptic.*—Perchloride of iron combined with collodion is a good hœmostatic for wounds, the bite of leeches, &c. To prepare it, one part of crystalized perchloride of iron should be added gradually and carefully to prevent the evolution of excessive heat, which injures the collodion. The composition, when well made, is of a yellowish red color, perfectly limpid, and produces on the skin a yellowish pellicle, which retains great elasticity.—*Ibid.*

